# Depressive Symptoms Are Associated with Linguistic Features of Negative but Not Positive Autobiographical Narratives

**DOI:** 10.1007/s42761-026-00395-4

**Published:** 2026-08-11

**Authors:** Alison B. Tuck, Yiqin Zhu, Renee J. Thompson

**Affiliations:** Department of Psychological & Brain Sciences, Washington University in St. Louis, CB 1125 One Brookings Drive, St. Louis, MO 63130-4899 USA

**Keywords:** Depression, Linguistics, Autobiographical, LIWC

## Abstract

Major depressive disorder (MDD) is associated with variation across several linguistic features, including more negative tone, less positive tone, greater use of first-person pronouns, and reduced use of affiliation words. This research has largely relied on analyzing texts from clinical samples, autobiographical reports of negatively valenced content, or unprompted narratives. This represents a significant gap in the literature, as large-scale language datasets (e.g., social media) increasingly capture individuals with depressive symptoms outside of MDD who express both positive and negative self-relevant content. However, whether linguistic features vary with depressive symptoms outside the context of MDD and whether these associations differ based on the emotional valence of the recalled content (i.e., positive vs. negative) remains unclear. In the present study, undergraduate students (*N* = 457) were randomly assigned to write about a sad or happy personal experience in as much detail as possible. We assessed negative tone, positive tone, first-person pronoun usage, and affiliation words using the Linguistic Inquiry and Word Count (LIWC) program. Greater depressive symptoms were associated with more negative tone and more first-person pronoun usage (i.e., greater self-focus) in the negative condition, as hypothesized, but depressive symptoms were not associated with linguistic features in the positive condition. Depressive symptoms did not relate to positive tone or use of affiliation words. Findings suggest that social-emotional biases present in depression may pertain to the processing of negative but not positive personal events and hold implications for identifying depressive symptoms in non-clinical and clinical settings and large linguistic databases.

**Supplementary Information:**

The online version contains supplementary material available at https://doi.org/10.1007/s42761-026-00395-4.

Major depressive disorder (MDD) is characterized by challenges in emotional and social functioning (Rottenberg & Gotlib, 2004). Researchers have harnessed computerized language models to identify linguistic markers of these challenges in people’s written texts, such as emotional tone and use of personal versus social words (Corbin et al., 2023). However, prior research has been constrained by a focus on clinical samples (e.g., individuals with MDD) and often on negatively valenced content. Whether these linguistic features generalize to depressive symptoms beyond MDD and emerge when individuals are prompted to recall negative *and* positive events is unclear. This has implications for understanding the social-emotional biases present in those with depressive symptoms (not just MDD) and for research seeking to assess depressive symptoms across different experiences (e.g., across social media content), where people may be compelled to write about a variety of experiences, including both negative and positive ones. Using an experimental design with college students, a population disproportionately affected by depression (Liu et al., 2019), we examined how depressive symptoms relate to social-emotional linguistic features in recollections of negative versus positive autobiographical events.

## Cognitive Biases in Depression

The psycholinguistic literature can be complemented by insights from cognitive psychology. Depression has been consistently linked with cognitive biases of negative information. For example, depression is associated with diminished reinforcement learning for symbols associated with low (but not high) reward probabilities (Vandendriessche et al., 2023). In addition, compared to their nondepressed peers, those with depression have been found to interpret negative and neutral visual stimuli as more negative (for reviews, see Huang et al., 2023; Peckham et al., 2010; Suslow et al., 2020). Notably, some have synthesized evidence of reduced positivity biases in depression as well (for review, see Suslow et al., 2020), whereas others have concluded that no such positivity bias is present (for review, see Huang et al., 2023). Together, there is robust evidence of a negativity bias in depression and some mixed evidence for a reduced positivity bias. However, unlike linguistic analyses, cognitive tasks cannot be easily harnessed to ascertain the extent to which individuals demonstrate biases in recalling their own negative and positive experiences (Fung et al., 2017). Only one study examined the trends in linguistic differences between positive and negative personal narratives among people with (*N* = 7) and without depression (*N* = 28; Herbert et al., 2019), whose small sample size prevented analytic conclusions (Herbert et al., 2019).

## Use of Clinical (versus Non-Clinical) Samples

Most research is focused on examining linguistic and cognitive biases in those with MDD. For instance, Himmelstein et al. (2018) examined linguistic features of autobiographical memories in a sample of 45 adults with MDD (compared to healthy controls), finding that MDD status was associated with greater “I” usage when recalling negative memories and less “I” usage when recalling positive memories. These findings suggest a negative cognitive bias for negative compared to positive memories, but studies such as these are limited to relatively small samples of people diagnosed with MDD, meaning results cannot be readily generalized to large numbers of people, including nonclinical samples who experience varying levels of depressive symptoms. Subthreshold depressive symptoms are ubiquitous across the U.S., with 18.5% of people over 18 experiencing depressive symptoms over the past two weeks (Villarroel & Terlizzi, 2020). College students are particularly vulnerable, with 35.5% of North American college students reporting depressive symptoms (Li et al., 2022). Given the prevalence of depressive symptoms in the population, it is critical to examine how depressive symptoms, not just MDD, relate to linguistics to gain a fuller and more generalizable picture of how psychological well-being manifests in language. College students may be the ideal population to examine linguistic trends due to the higher prevalence of depressive symptoms in this group.

## Broad Linguistic Features in Depression

Affective abnormalities in depression are present across several response systems (experiential, behavioral, and physiological; Bylsma et al., 2008) and extend to language. For instance, Stamatis et al. (2022) found that increased depressive symptoms over time were associated with less language about social processes and affiliation words in participants’ text messages over the same time period. Studies such as this help shed insights into how depressive symptoms can be detected in unstructured, unprompted language and provide a better understanding of the constructs that characterize depression (e.g., social withdrawal).

Additional studies have examined how depression relates to language when discussing difficult topics. For instance, in studies examining linguistic features within Reddit posts to mental health communities (Silveira et al., 2021) and among undergraduates writing about their transition to college (Rude et al., 2004), depression was linked with more negative and less positive emotional tone. Notably, this research relies on writing about challenging topics that are likely to induce negative experiences.

Several studies have broadly utilized language from social media to detect mental health features of social media users. For example, these studies have identified that (a) university students frequently disclose symptoms of depression, anxiety, stress, suicidal ideation, and psychosis on their university’s sub-Reddit pages (Saha et al., 2022) and (b) depressive symptoms can be detected in Facebook users’ language referencing predictors of depression such as sadness, loneliness, hostility, and rumination (Eichstaedt et al., 2018). This research broadly underscores the powerful role of natural language to predict depression in online communities. However, people often attempt to describe positive experiences when wanting to make positive impressions on others and build interpersonal trust and intimacy (Reis et al., 2010). This may be especially true on social media, where research shows people are inclined to present positive information about themselves to present positive social impressions (see Hollenbaugh, 2021, for a review). To fully understand the social-emotional biases underlying depression and properly recognize depression in written text, it is critical to determine whether depressive symptoms are linked to linguistic markers in positive descriptions.

## What We Know and Do Not Know about Specific Language Features of Depression

Several features of language provide insights into the social-emotional biases present in those with elevated depressive symptoms. Positive and negative emotional tone represents language summaries that reflect people’s overall sentiment conveyed in written language (Boyd et al., 2022). Other language features are thought to convey specific psychological constructs such as attentional focus on constructs that words represent (Boyd & Schwartz, 2021). First-person pronoun usage reflects attention on oneself (e.g., “me,” “my,” or “I”; Ashokkumar & Pennebaker, 2022). Increased use of first-person pronouns is robustly linked with depression (see Edwards & Holtzman, 2017, for a review). However, one seminal study by Pyszczynski and Greenberg (1987) found that, in verbal interviews with people with depression, they demonstrated more self-focused attribution bias following negative events but not positive events. It is unclear whether this ruminative self-fixation can be detected in people’s *written* texts between negative and positive experiences, which could shed insights into biased cognitive processing across valence and in writing.

While first-person pronoun use reflects a focus on oneself, affiliation words reflect a person’s attention on others (e.g., “we”, “together”; Ashokkumar & Pennebaker, 2022). Evidence suggests depression is linked to reduced use of affiliation words in unprompted language (i.e., text messages to others; Meyerhoff et al., 2023). These findings are sensible given social withdrawal in depression (Wolters et al., 2023), meaning those with depression may not be as attentive to social constructs. Overall, psycholinguistic literature about affiliation words in depression is small, and to gain further insights into social deficits in depression, it is important to examine whether language reflecting reduced attention on others is present even when people recall positive experiences, which are often more social in nature than negative experiences (Arewasikporn et al., 2019).

## The Current Study

The current study represents a replication of Himmelstein et al. (2018) and Herbert et al. (2019). In addition, it extends prior research by examining how depressive symptoms (beyond MDD diagnoses) relate to social-emotional linguistic features in written recollections of negative and positive autobiographical events. Specifically, we used linguistic analysis to examine emotional tone (i.e., negative and positive tone) and self versus other attention (i.e., first-person pronoun usage versus affiliation words) related to college students’ depressive symptoms when they recalled negative or positive autobiographical events. Given depression’s link with cognitive biases for negative (more so than positive) content, we hypothesized that depressive symptoms would relate to elevated negative tone and reduced positive tone in the negative (but not positive) condition. Given some evidence of discounting positives in depression, we hypothesized depressive symptoms would be associated with more first-person pronoun usage when recalling negative events and less first-person pronoun usage when recalling positive events. Given social disconnection in depression, we hypothesized that depressive symptoms would relate to fewer affiliation words in both conditions (i.e., no interaction effect).

## Method

### Participants

The original sample consisted of 462 university students, and the final analytic sample included 457 participants (98.9%) of the original sample. Please see **Linguistic Feature Extraction** for details about exclusion. Participants ranged in age from 18 to 22 (*M*_*age*_ = 19.2, *SD*_*age*_ = 1.2), and 64.1% of participants identified as women, 35.7% identified as men, and 0.2% did not disclose. The racial demographics were as follows: 54.29% White, 26.37% Asian, 10.11% African American or Black, 8.35% Multiracial, and 0.44% American Indian or Alaska Native. Sample size was determined based on a prior power analysis for the parent investigation focused on examining how college students’ emotions before using social media related to the emotional effects of their real-time social media use (Tuck et al., 2023).

### Procedures

Undergraduate students at a Midwest university were recruited through the university’s research subject pool. Study procedures took place online via a pre-programmed Qualtrics survey. After participants completed informed consent, they were navigated to a page with experimental instructions. Procedures were approved by the Washington University in St. Louis institutional review board (protocol #202004076). Informed consent was obtained from all participants included in the study. Upon completion, participants were compensated with course research credit.

**Emotional induction procedure.** These procedures were adapted from a previous study examining the best methods for inducing emotion states (Ribeiro et al., 2019). Participants were randomly assigned to either a sad (*n* = 228) or a happy (*n* = 229) emotion condition. Among the 457 participants, the regression models included 453 complete cases because four had missing MASQ-AD data. We utilized a between-person design due to time limitations and a need to minimize participant burden. Participants listened to mood-congruent music while writing for three minutes. Following recommendations from Zhang et al. (2014), participants in the sad induction listened to a segment of Adagio in G Minor composed by Remo Giazotto (1958), while those in the happy condition listened to a segment of Brandenburg Concerto No. 2 in F Major composed by Johann Sebastian Bach (1721). They received these instructions: “Next, you will be listening to music while writing about the most SAD [HAPPY] past experience you can think of. You do not need to write well or even in complete sentences. Reexperience the event as vividly as possible. Please think about how you felt at the time the event occurred, what led to these feelings, and whether the event elicited thoughts or fantasies that increased their bad [good] feeling.” These methods successfully increased positive affect and decreased negative affect in the happy condition and increased negative affect and decreased positive affect in the sad condition (Tuck et al., 2023), measured by participants’ scores on the Positive and Negative Affect Schedule-20 (PANAS-20; Watson et al., 1988). Since neither happiness nor sadness is included as a discrete affect variable in the PANAS-20, we could not assess changes in happiness or sadness.

### Measures

**Linguistic Feature Extraction.** We used the Linguistic Inquiry and Word Count, 2022 (LIWC-2022) program (Boyd et al., 2022) to assess language in participants’ autobiographical narratives. Research suggests that language-based predictions of individual characteristics (e.g., personality or age) showed increased accuracy with a greater number of words (Kern et al., 2016). To reduce the influence of very short, sparse texts while balancing the sample to be retained, we excluded narratives that were 40 words or fewer from analyses^1^. Four linguistic features (positive tone, negative tone, I-usage, and affiliation words) that assessed our constructs of interest (positive emotional tone, negative emotional tone, attention on oneself, and need to connect with others) were extracted from individuals’ autobiographical narratives by the LIWC program (Boyd et al., 2022). The dictionary development and validation procedures showed that these four features demonstrate excellent internal consistencies (KR-20 ranged from 0.85 to 0.98; Boyd et al., 2022). Those procedures derived the internal consistencies by treating individual words within a linguistic feature’s dictionary as items (e.g., presence/absence of each word). In the current sample, internal consistencies were not computable because LIWC produced a single score for each linguistic feature, and the item-level information was not available.

**Depressive Symptoms.** We administered the Anhedonic Depression scale from the Mood and Anxiety Symptom Questionnaire (MASQ-AD; Watson et al., 1995). The MASQ-AD contains 22 items assessing symptoms over the past week from 1 (*not at all*) to 5 (*extremely*). Items are summed to compute a total depression score. Due to ethical considerations associated with the study taking place online, the suicidal ideation item was excluded. This scale has been validated with college student samples (Watson et al., 1995), and the internal consistency of items in the current sample was excellent (*α* = 0.93).

## Analytic Plan

All analyses were conducted in R (version 4.5.2), and linear models were estimated using the base lm() function. We conducted a post-hoc power analysis using the pwr package in R to determine the smallest detectable effect size given the sample size. Assuming *α* = 0.05 and power = 0.80, the complete-case analytic sample (with 448 degree of freedom) provided adequate power to detect small effects (f² = 0.017; ~1.7% variance explained) for an individual predictor. Before analysis, outliers (i.e., over three standard deviations) were winsorized to three standard deviations from the means for each variable. We first compared any differences in key variables (demographic, depression severity, word count, and four linguistic features) in the two conditions. Specifically, we used independent-samples *t*-tests for continuous variables (e.g., age, depression severity, and word count), and chi-square tests were used for categorical variables (gender, race, and ethnicity). Then, within each condition, we examined the zero-order correlations between depression severity and each linguistic feature.

Four separate linear regression models were performed for each linguistic feature (i.e., positive tone, negative tone, self-focus, and affiliation). Each model included three predictors: the condition (positive vs. negative), depressive symptoms, and a condition by depressive symptoms interaction. Analyses indicated that word count was not associated with depressive symptoms, induction condition, or their interaction and thus was not included as a covariate in models. To retrieve simple slopes of depression within each condition and their significance, we recoded the condition variables (i.e., switching the reference group) and re-ran models.

## Results

### Descriptive and Zero-Order Correlations

Table 1 presents descriptive statistics of demographic, clinical, and linguistic features, group differences in all variables of interest, and zero-order correlations between depression severity and linguistic features within each condition. Results indicated that there were no significant differences in depression severity (*t* = 0.002; *df =* 448.67, *p* = .998), the number of words (*t = 1.57*,* df = 451.02*, *p = .118*), the gender distribution (𝜒^2^(1) = 0.34, *p* = .558), or racial distribution (𝜒^2^(3) = 8.39, *p* = .078) between the positive and negative writing conditions. However, individuals in the positive condition were older (*M* = 19.3 years; *SD* = 1.2 years) than those in the negative condition (*M* = 19.1 years, *SD* = 1.1 years, *t = 2.10*,* df = 450.26*, *p = .036*). As a manipulation check, texts in the positive condition showed greater positive tone (*t* = 17.60, *df* = 411.99, *p* < .001) and weaker negative tone (*t* = -18.47, *df* = 306.44, *p* < .001) than texts in the negative condition.

**Table 1:** Descriptive statistics by the conditions of two autobiographical narratives in the eligible sample with narratives exceeding 40 words (*N* = 457)

	Positive (*N* = 229)		Negative (*N* = 228)		Condition difference
	*M* (*SD*)		*M* (*SD*)		(t or 𝜒^2^)
Age	19.3 (1.2)		19.1 (1.1)		*t =* 2.10, *df* = 450.26, *p =* .036
Range	17–22		17–22		
Gender					𝜒^2^(1) = 0.34, *p =* .558
Female	143 (62.4%)		150 (65.8%)		
Male	85 (37.1%)		78 (34.2%)		
Other/Unknown	1 (0.4%)		0 (0%)		
Race					𝜒^2^(4) = 8.39, *p =* .078
White	109 (48.0%)		138 (60.5%)		
Black or African American	24 (10.6%)		22 (9.7%)		
Asian	72 (31.7%)		48 (21.1%)		
Multiracial	20 (8.8%)		18 (7.9%)		
American Indian or Alaska Native	2 (0.9%)		2 (0.9%)		
Other/Unknown	2 (0.9%)		0 (0%)		
Ethnicity					𝜒^2^(1) = 0.33, *p =* .564
Hispanic/Latino	28 (12.2%)		23 (10.1%)		
Depression Severity					
MASQ AD	63.3 (13.6)		63.3 (14.6)		*t =* 0.002, df = 448.67, *p =* .998
Linguistic Features	*M* (*SD*)	Cor with MASQ-AD	*M* (*SD*)	Cor with MASQ-AD	Condition difference
Word Count	125.4 (39.0)	*r* = − .002, *p* = .973	119.9 (35.3)	*r* = .016, *p* = .811	*t =* 1.57, *df* = 451.02, *p =* .118
Positive Tone	5.30 (2.33)	*r* = − .104, *p* = .117	1.88 (1.72)	*r* = .003, *p* = .961	*t =* 17.87, *df* = 411.99, *p* < .001
Negative Tone	0.78 (0.97)	*r* = .033, *p* = .619	3.78 (2.21)	*r* = .172, *p* = .010	*t =* -18.78, *d*f = 306.44, *p* < .001
I-usage	8.26 (3.97)	*r* = − .047, *p* = .484	10.54 (3.94)	*r* = .152, *p* = .022	*t =* -6.17, *df* = 454.97, *p* < .001
Affiliation	4.24 (2.87)	*r* = − .114, *p* = .088	3.38 (2.46)	*r* = − .010, *p* = .877	*t =* 3.43, *df* = 442.64, *p* < .001

*MASQ-AD* the Mood and Anxiety Symptoms Questionnaire, Anhedonic Depression Scale

Bivariate associations showed that in the positive condition, depression severity was not significantly associated with any linguistic features (*r*s: − 0.11 to 0.03; *df* = 227; *p*s > 0.05). Within the negative condition, depression severity was associated with greater negative tone (*r* = .17; *df* = 226; *p* = .001) and self-focus (*r* = .15; *df* = 226; *p* = .02), whereas no associations were observed for positive tone (*r* = .003; *df* = 226; *p* = .961) or affiliation (*r* = − .01; *df* = 226; *p* = .877).

### Main Effects of Condition in Regression Models

The main effects of the condition on linguistic features are presented in Table 2. Supporting the effectiveness of the induction, individuals in the positive condition wrote narratives that had a more positive tone than those in the negative condition (𝛽 = 0.84, 95% CI = [0.51, 1.17], *p* < .001), and individuals in the negative condition wrote narratives with more negative tone than those in the positive condition (𝛽 = 0.33, 95% CI = [0.01, 0.64], *p* = .045). Further, individuals wrote narratives with more affiliation words in the positive condition compared to the negative condition (𝛽 = 0.42, 95% CI = [0.001, 0.84], *p* = .049). No differences emerged in I-usage between the two conditions (𝛽 = 0.14, 95% CI = [-0.26, 0.55], *p* = .498).

**Table 2:** Linguistic features predicted by severity of depression, induction conditions (positive vs. negative), and their interaction (*N* = 453; retaining responses over 40 words). Significant findings were presented in bold type

Linguistic feature	Predictors	Hypothesis	Results				
		Direction of effect	*R*^2^	b	𝛽	95% CI of 𝛽	*p*
Positive Tone	**Intercept**	**n/a**	**0.40**	**1.87**	**n/a**	**n/a**	**0.003**
	**Condition**	**Positive > Negative**		**4.57**	**0.84**	**[0.51**,** 1.17]**	**< 0.001**
	Depression (Condition=negative)	*ß*_Neg_ < 0		0.0004	0.00	[-0.10, 0.10]	0.968
	Depression (Condition=positive)	*ß*_Pos_ < 0		-0.02	-0.09	[-0.20, 0.01]	0.087
	Depression*Condition	*ß*_Neg_ < *ß*_Pos_ < 0		-0.02	-0.22	[-0.56, 0.12]	0.202
Negative Tone	**Intercept**	**n/a**	**0.45**	**2.12**	**n/a**	**n/a**	**< 0.001**
	**Condition**	**Positive < Negative**		**-1.50**	**-0.33**	**[-0.64**,** -0.01]**	**0.045**
	**Depression (Condition=negative)**	***ß***_**Neg**_ **> 0**		**0.03**	**0.16**	**[0.07**,** 0.26]**	**< 0.001**
	Depression (Condition=positive)	*ß*_Pos_ > 0		0.002	0.01	[-0.09, 0.12]	0.778
	Depression*Condition	***ß***_**Neg**_ **>** ***ß***_**Pos**_ **> 0**		**-0.02**	**-0.35**	**[-0.67**,** -0.02]**	**0.036**
I-usage	**Intercept**	**n/a**	**0.09**	**7.91**	**n/a**	**n/a**	**< 0.001**
	Condition	Positive < Negative		1.17	0.14	[-0.26, 0.55]	0.498
	**Depression (Condition=negative)**	***ß***_**Neg**_ **> 0**		**0.04**	**0.14**	**[0.02**,** 0.26]**	**0.022**
	Depression (Condition=positive)	*ß*_Pos_ < 0		-0.01	-0.05	[-0.18, 0.08]	0.477
	Depression*Condition	***ß***_**Pos**_ **< 0 <** ***ß***_**Neg**_		**-0.05**	**-0.44**	**[-0.86**,** -0.03]**	**0.037**
Affiliation Words	**Intercept**	**n/a**	**0.03**	**3.52**	**n/a**	**n/a**	**< 0.001**
	**Condition**	**Positive > Negative**		**2.32**	**0.42**	**[0.001**,** 0.84]**	**0.049**
	Depression (Condition=negative)	*ß*_Neg_ < 0		-0.002	-0.01	[-0.13, 0.12]	0.888
	Depression (Condition=positive)	*ß*_Pos_ < 0		-0.02	-0.13	[-0.26, 0.01]	0.064
	Depression*Condition	*ß*_Neg_ = *ß*_Pos_		-0.02	-0.27	[-0.70, 0.15]	0.209

Sensitivity analyses controlling for age showed the same pattern of results for each analysis, with minimal differences in effect sizes with or without age. See Table S2 in the supplemental materials for detailed results

### Linguistic Features Predicted by Depressive Symptoms by Condition

**Fig. 1:**
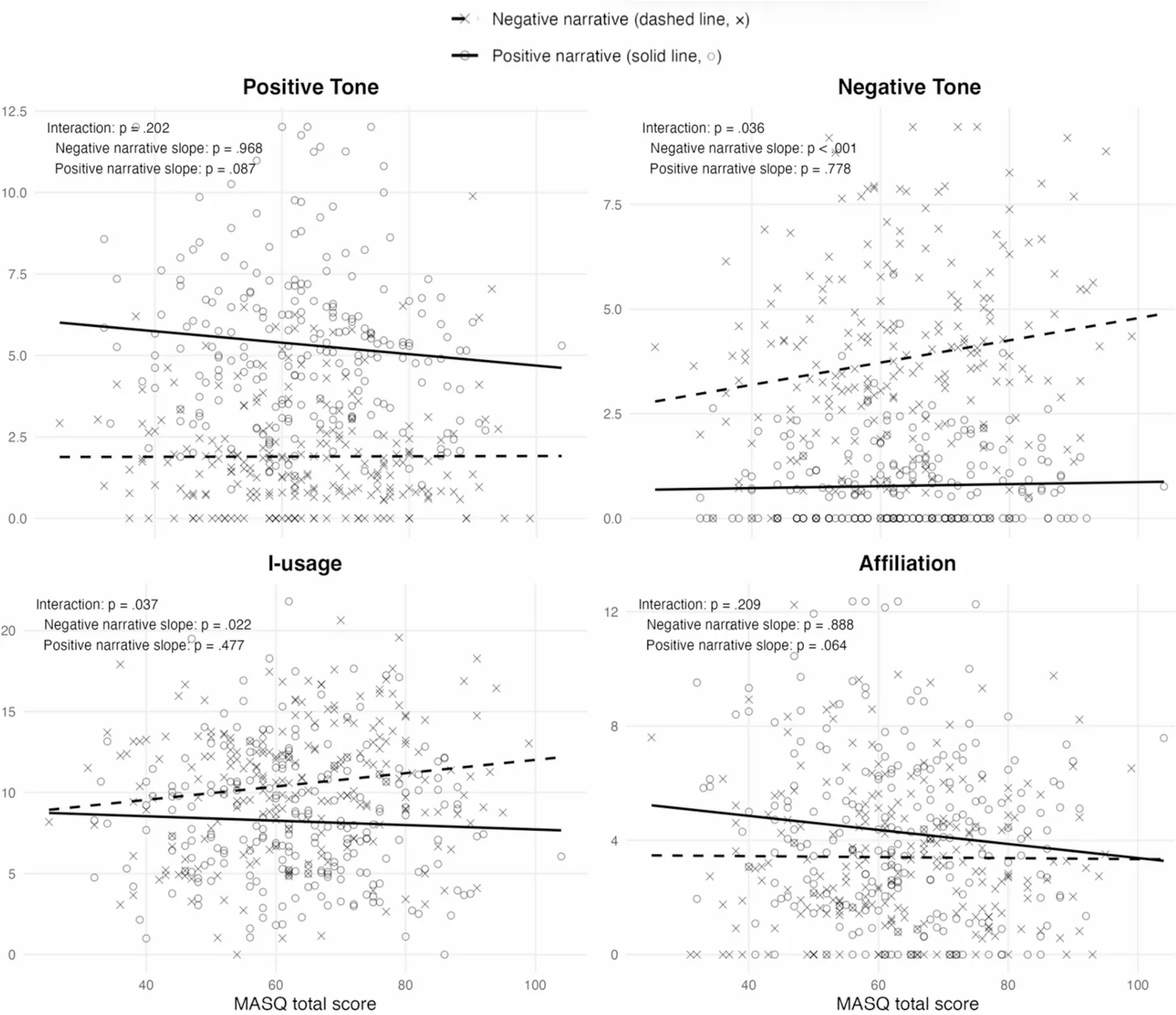
Associations between depression symptoms (MASQ-AD) and linguistic features in negative and positive narratives (*N* = 453). Solid lines with open circles represent the positive narrative condition; dashed lines with x-shaped markers represent the negative narrative condition. Panel annotations display p-values. The associations between depression symptoms and negative tone and I-usage in the negative narrative condition, but not in the positive narrative condition, were significant

Linear regression results for how condition moderated depressive symptoms’ associations with linguistic features are presented in Table 2 and Fig. 1. Consistent with hypotheses, condition moderated the associations between negative tone and depression (𝛽 = -0.35, *p* = .036). Simple slopes indicated that the associations between depression and negative tone were stronger in the negative condition than in the positive condition. Specifically, individuals with higher depression severity used more negative tone in the negative condition (𝛽 = 0.14, *p* = .022), but not in the positive condition (𝛽 = 0.002, *p* = .778). Consistent with hypotheses, condition moderated the associations between I-usage and depression (𝛽 = -0.44, *p* = .037), and the association between I-usage and depression was stronger in the negative condition compared to the positive condition. Specifically, individuals with higher depression severity used more negative tone in the negative condition (𝛽 = 0.14, *p* = .022). However, individuals with higher depression did not use more negative tone in the positive condition (𝛽 = -0.05, *p* = .477). Inconsistent with the hypotheses, condition did not moderate the associations between depression and positive tone (𝛽 = -0.22, *p* = .202); in both groups, depression severity was not significantly associated with positive tones (Negative: 𝛽 = 0.00, *p* = .968; Positive: 𝛽 = -0.09, *p* = .087) without significant differences in effect sizes. Although, as expected, condition did not moderate the associations between depression and affiliation usage (𝛽 = -0.27, *p* = .209), suggesting non-significant differences in the effect sizes in the two conditions. For both condition groups, there were no significant associations between depression and affiliation word usage (Negative: 𝛽 = -0.01, *p* = .888; Positive: 𝛽 = -0.13, *p* = .064).

## Discussion

This investigation utilized an emotion induction paradigm to examine features of language present in positive and negative autobiographical narratives, with a particular focus on how depressive symptoms moderated these associations. College students’ depressive symptoms were related to more negative tone and language reflecting attention on oneself in the negative emotion condition but were not associated with linguistic features in the positive condition. Notably, the effect sizes for the significant associations in the negative conditions were small but comparable to those in psycholinguistic research, including meta-analytic results on depression and I-usage (Holtzman, 2017) and linguistic correlates to personality traits (Kern et al., 2014). The significant interactions were fairly robust, showing moderate effect sizes.

These findings hold important implications for informing our understanding of social-emotional processing in those experiencing depressive symptoms. Our results suggest that the negative emotional and self-focused biases known to characterize depression (Pyszczynski et al., 1989) may only be detectable when people recount negative experiences. This conceptually supports theory from cognitive psychology, indicating that depression may relate to cognitive biases for negative (but not positive) information that is not personally relevant (e.g., Vandendriessche et al., 2023). Since emotional and social functioning are likely to be more impaired among people with MDD, it is possible that findings from this investigation would be further exacerbated in the written text of negative narratives in clinical samples.

Importantly, our findings suggest that linguistic features associated with emotional and self-focused biases are detectable in a non-clinical college student sample. This highlights two related implications: (a) emotional and self-focused biases of negative experiences may characterize the experience of depressive symptoms broadly and not only depressive disorders, and (b) these biases can likely be detected in the written language of non-clinical samples. This points to the potential power of linguistic analyses to characterize and detect symptoms of mental health disorders in non-clinical samples.

Relatedly, these findings are important to consider when examining depressive symptoms via linguistic analyses from large online databases like social media. Although research suggests depressive symptoms are detectable via people’s self-disclosures of symptoms on social media (e.g., Salas-Zárate et al., 2022), depression is also associated with an increased likelihood of posting positive social media texts that present favorable social impressions (Nesi & Prinstein, 2015; Tuck et al., 2025). Our findings revealed that depressive symptoms were not associated with linguistic features in the positive condition. Thus, it may be more difficult to detect depressive symptoms on social media than researchers previously believed (e.g., Godara et al., 2023). Future research could explore and characterize the linguistic features of positive texts written by those with depression to identify which features of language, if any, are related to symptoms in this context. Positive social media content may be identified based on factors such as positive text tone (i.e., sentiment analysis), the use of positive words, or visually positive images. Improved detectability of depressive symptoms based on negative *and* positive social media content via language could also raise the possibility of implementing technology-assisted health interventions in these spaces, such as sending targeted psychoeducation about depression via social media.

Clinically, these findings point to the possibility that linguistic markers might be an effective monitoring tool for depressive symptoms. Writing about negative experiences is a common therapeutic tool used to help clients process information (Asmundson et al., 2019). More direct research is needed to test whether clients’ writing becomes less/more negative and less/more self-focused when their depression improves/worsens. These cognitive changes may not be easily detectable by the therapist or the clients themselves, but linguistic analysis tools can readily identify them. This objective indicator of symptom change could therefore be used in combination with client self-reports, and future research should directly examine this possibility.

Due to the study design, it is unclear whether the associations between depressive symptoms and linguistic markers were due to participants writing about something negative, or due to negative mood, or both. To disentangle this, research is needed to test whether these effects characterize people’s spontaneous writings about negative experiences outside the context of mood induction. Although we cannot directly answer this question with our data, we ran a post hoc analysis, finding that depressive symptoms interacted with the negative condition to predict more negative tone and first-person pronouns, controlling for change in NA and PA during the emotion induction (Supplemental Materials, Table S3). This suggests observed findings were not driven by emotions experienced from the induction and were instead driven by the valence of the autobiographical event.

Future research would benefit from examining whether findings from the current investigation are specific to having depressive symptoms specifically or whether they generalize to the experience of negative emotionality, various internalizing symptoms, or emotion dysregulation more broadly. For instance, since emotional tone is thought to reflect current mood (Eichstaedt & Weidman, 2020), those broadly susceptible to negative mood states may convey heightened negative tone when recalling negative events, whether heightened negative mood can be attributable to depression, anxiety, or general temperament (e.g., high neuroticism). Further, we capitalized on a young adult sample due to their high prevalence of depression, but future investigations should assess these linguistic features in middle- and older-adult samples. Individuals in old age experience higher emotional well-being (Charles & Carstensen, 2010) and show lower prevalence rates of MDD (Hasin et al., 2005) compared to younger adults. Given different emotional experiences across developmental stages, it is important to ensure that the results generalize across the age range. Finally, emerging evidence suggests that race moderates the relationship between language features extracted from social media and depression; “I” usage on social media was associated with depression in White but not Black individuals, and machine learning models trained to predict depression severity from social media language performed poorly for Black but not White participants (Rai et al., 2024). This research suggests that the models the field relies upon to characterize the linguistic features of depression may adequately capture White people’s experiences while failing to adequately capture the experiences of Black individuals and possibly those from other racial backgrounds. As almost half of participants in the current study were non-White, results may need to be interpreted with caution. It will be critical for future research to continue examining how linguistic features vary as a function of race in order to develop linguistic models that can reliably characterize and represent all people.

Linguistic analysis is a powerful tool for examining people’s emotional and social processes. By capitalizing on this tool and an experimental design, we found social and emotional biases in autobiographical accounts related to depressive symptoms during negative, but not positive, narratives in a non-clinical sample of college students. These findings hold important implications for (a) understanding how social-emotional biases are associated with depressive symptoms in non-clinical samples and (b) gaining clarity and confidence in the field’s ability to detect these biases in large publicly available language datasets (e.g., social media) where researchers are turning more readily to access population-level data to answer some of the most salient questions facing today’s technology-forward society.

## Electronic Supplementary Material

Below is the link to the electronic supplementary material.

Supplemental Materials

## Footnotes

1 *We ran the four linear models using texts that contained over 25 words based on Boyd (2017). Results are presented in Table S1. The pattern of results was consistent across the 25- and 40-word thresholds*,* with minimal differences in effect sizes.*

## Additional Information

### Data Availability

Data will be available upon reasonable request to the corresponding author. Analysis scripts for this study have been made openly available at https://osf.io/skmh6/?viewonly=9ac57b830f4849ae9f885a25d326bfa7.

## References

[CR1] Arewasikporn, A., Sturgeon, J. A., & Zautra, A. J. (2019). Sharing positive experiences boosts resilient thinking: Everyday benefits of social connection and positive emotion in a community sample. *American Journal of Community Psychology*, *63*(1–2), 110–121. 10.1002/ajcp.12279 30295327 PMC6405300

[CR2] Ashokkumar, A., & Pennebaker, J. W. (2022). Tracking group identity through natural language within groups. *PNAS Nexus*, *1*(2), pgac022. 10.1093/pnasnexus/pgac022 35774418 PMC9229362

[CR3] Asmundson, G. J., Thorisdottir, A. S., Roden-Foreman, J. W., Baird, S. O., Witcraft, S. M., Stein, A. T., & Powers, M. B. (2019). A meta-analytic review of cognitive processing therapy for adults with posttraumatic stress disorder. *Cognitive Behaviour Therapy*, *48*(1), 1–14. 10.1080/16506073.2018.1522371 30332919

[CR5] Boyd, R. L., & Schwartz, H. A. (2021). Natural language analysis and the psychology of verbal behavior: The past, present, and future states of the field. *Journal of Language and Social Psychology*, *40*(1), 21–41. 10.1177/0261927X20967028 34413563 PMC8373026

[CR4] Boyd, R. L., Ashokkumar, A., Seraj, S., & Pennebaker, J. W. (2022). The development and psychometric properties of LIWC-22. *Austin, TX: University of Texas at Austin, 10*. 10.13140/RG.2.2.23890.43205

[CR6] Bylsma, L. M., Morris, B. H., & Rottenberg, J. (2008). A meta-analysis of emotional reactivity in major depressive disorder. *Clinical Psychology Review*, *28*(4), 676–691. 10.1016/j.cpr.2007.10.001 18006196

[CR7] Charles, S. T., & Carstensen, L. L. (2010). Social and emotional aging. *Annual Review of Psychology*, *61*(1), 383–409. 10.1146/annurev.psych.093008.100448 19575618 PMC3950961

[CR8] Corbin, L., Griner, E., Seyedi, S., Jiang, Z., Roberts, K., Boazak, M., Bahrami Rad, A., Clifford, G. D., & Cotes, R. O. (2023). A comparison of linguistic patterns between individuals with current major depressive disorder, past major depressive disorder, and controls in a virtual, psychiatric research interview. *Journal of Affective Disorders Reports*, *14*, 100645. 10.1016/j.jadr.2023.100645

[CR9] Edwards, T., & Holtzman, N. S. (2017). A meta-analysis of correlations between depression and first person singular pronoun use. *Journal of Research in Personality*, *68*, 63–68. 10.1016/j.jrp.2017.02.005

[CR11] Eichstaedt, J. C., & Weidman, A. C. (2020). Tracking fluctuations in psychological states using social media language: A case study of weekly emotion. *European Journal of Personality*, *34*(5), 845–858. 10.1002/per.2261

[CR10] Eichstaedt, J. C., Smith, R. J., Merchant, R. M., Ungar, L. H., Crutchley, P., Preoţiuc-Pietro, D., & Schwartz, H. A. (2018). Facebook language predicts depression in medical records. *Proceedings of the National Academy of Sciences*, *115*(44), 11203–11208. 10.1073/pnas.1802331115 PMC6217418 30322910

[CR12] Fung, C. K., Moore, M. M., Karcher, N. R., Kerns, J. G., & Martin, E. A. (2017). Emotional word usage in groups at risk for schizophrenia-spectrum disorders: An objective investigation of attention to emotion. *Psychiatry Research*, *252*, 29–37. 10.1016/j.psychres.2017.01.098 28242515 PMC5438895

[CR13] Godara, R. K., Mengi, A., Sharma, A., & Sharma, S. (2023). Detecting depressive symptoms on social media: A comprehensive review of methodologies and strategies for suicide prevention. In *international conference on cognitive computing and cyber physical systems* (pp. 87–100). Singapore: Springer Nature Singapore.

[CR14] Hasin, D. S., Goodwin, R. D., Stinson, F. S., & Grant, B. F. (2005). Epidemiology of major depressive disorder: Results from the National Epidemiologic Survey on Alcoholism and Related Conditions. *Archives of General Psychiatry*, *62*(10), 1097–1106. 16203955 10.1001/archpsyc.62.10.1097

[CR15] Herbert, C., Bendig, E., & Rojas, R. (2019). My sadness–our happiness: Writing about positive, negative, and neutral autobiographical life events reveals linguistic markers of self-positivity and individual well-being. *Frontiers in Psychology*, *9*, 2522. 10.3389/fpsyg.2018.02522 30670993 PMC6331680

[CR16] Himmelstein, P., Barb, S., Finlayson, M. A., & Young, K. D. (2018). Linguistic analysis of the autobiographical memories of individuals with major depressive disorder. *PloS One*, *13*(11), e0207814. 10.1371/journal.pone.0207814 30475918 PMC6258120

[CR17] Hollenbaugh, E. E. (2021). Self-presentation in social media: Review and research opportunities. *Review of Communication Research, 9*, 80–98. 10.12840/ISSN.2255-4165.027

[CR18] Holtzman, N. S. (2017). A meta-analysis of correlations between depression and first person singular pronoun use. *Journal of Research in Personality*, *68*, 63–68. 10.1016/j.jrp.2017.02.005. https://doi-org.libproxy.washu.edu/

[CR19] Huang, G., Li, Y., Zhu, H., Feng, H., Shen, X., & Chen, Z. (2023). Emotional stimulation processing characteristics in depression: Meta-analysis of eye tracking findings. *Frontiers in Psychology*, *13*, 1089654. 10.3389/fpsyg.2022.1089654 36710847 PMC9880408

[CR20] Kern, M. L., Eichstaedt, J. C., Schwartz, H. A., Dziurzynski, L., Ungar, L. H., Stillwell, D. J., Kosinski, M., Ramones, S. M., & Seligman, M. E. (2014). The online social self: An open vocabulary approach to personality. *Assessment*, *21*(2), 158–169. 10.1177/1073191113514104 24322010

[CR21] Kern, M. L., Park, G., Eichstaedt, J. C., Schwartz, H. A., Sap, M., Smith, L. K., & Ungar, L. H. (2016). Gaining insights from social media language: Methodologies and challenges. *Psychological Methods*, *21*(4), 507–525. 10.1037/met0000091 27505683

[CR22] Li, W., Zhao, Z., Chen, D., Peng, Y., & Lu, Z. (2022). Prevalence and associated factors of depression and anxiety symptoms among college students: a systematic review and meta-analysis. *Journal of Child Psychology and Psychiatry*, *63*(11), 1222–1230. 10.1111/jcpp.13606 35297041

[CR23] Liu, Y., Zhang, N., Bao, G., Huang, Y., Ji, B., Wu, Y., Liu, C., & Li, G. (2019). Predictors of depressive symptoms in college students: A systematic review and meta-analysis of cohort studies. *Journal of Affective Disorders*, *244*, 196–208. 10.1016/j.jad.2018.10.084 30352363

[CR24] Meyerhoff, J., Liu, T., Stamatis, C. A., Liu, T., Wang, H., Meng, Y., Curtis, B., Karr, C. J., Sherman, G., Ungar, L. H., & Mohr, D. C. (2023). Analyzing text message linguistic features: Do people with depression communicate differently with their close and non-close contacts? *Behaviour Research and Therapy*, *166*, 104342. 10.1016/j.brat.2023.104342 37269650 PMC10330918

[CR25] Nesi, J., & Prinstein, M. J. (2015). Using social media for social comparison and feedback-seeking: Gender and popularity moderate associations with depressive symptoms. *Journal of Abnormal Child Psychology*, *43*(8), 1427–1438. 10.1007/s10802-015-0020-0 25899879 PMC5985443

[CR26] Peckham, A. D., McHugh, R. K., & Otto, M. W. (2010). A meta-analysis of the magnitude of biased attention in depression. *Depression and Anxiety*, *27*(12), 1135–1142. 10.1002/da.20755 21049527

[CR27] Pyszczynski, T., & Greenberg, J. (1987). Toward an integration of cognitive and motivational perspectives on social inference: A biased hypothesis-testing model. *Advances in Experimental Social Psychology*, *20*, 297–340. 10.1016/S0065-2601(08)60417-7

[CR47] Pyszczynski,T., Hamilton, J. C., Herring, F. H., & Greenberg, J. (1989). Depression, self-focused attention, and the negative memory bias. *Journal of Personality and Social Psychology*, *57*(2), 351–357. 10.1037/0022-3514.57.2.351

[CR28] Rai, S., Stade, E. C., Giorgi, S., Francisco, A., Ungar, L. H., Curtis, B., & Guntuku, S. C. (2024). Key language markers of depression on social media depend on race. *Proceedings of the National Academy of Sciences, 121*(14), e2319837121. 10.1073/pnas.2319837121 PMC10998627 38530887

[CR29] Reis, H. T., Smith, S. M., Carmichael, C. L., Caprariello, P. A., Tsai, F. F., Rodrigues, A., & Maniaci, M. R. (2010). Are you happy for me? How sharing positive events with others provides personal and interpersonal benefits. *Journal of Personality and Social Psychology*, *99*(2), 311–329. 10.1037/a0018344 20658846

[CR30] Ribeiro, F. S., Santos, F. H., Albuquerque, P. B., & Oliveira-Silva, P. (2019). Emotion induction through music: Measuring cardiac and electrodermal responses of emotional states and their persistence. *Frontiers in Psychology*, *10*. 10.3389/fpsyg.2019.00451 PMC6414444 30894829

[CR31] Rottenberg, J., & Gotlib, I. H. (2004). Socioemotional functioning in depression. In M. Power (Ed.),*Mood Disorders: A Handbook of Science and Practice* (pp. 61–77). John Wiley & Sons.

[CR32] Rude, S., Gortner, E. M., & Pennebaker, J. (2004). Language use of depressed and depression-vulnerable college students. *Cognition and Emotion*, *18*(8), 1121–1133. 10.1080/02699930441000030

[CR33] Saha, K., Yousuf, A., Boyd, R. L., Pennebaker, J. W., & De Choudhury, M. (2022). Social media discussions predict mental health consultations on college campuses. *Scientific Reports*, *12*(1), 123. 10.1038/s41598-021-03423-4 34996909 PMC8741988

[CR34] Salas-Zárate, R., Alor-Hernández, G., Salas-Zárate, M. P., Paredes-Valverde, M. A., Bustos-López, M., & Sánchez-Cervantes, J. L. (2022). Detecting depression signs on social media: A systematic literature review. *Healthcare*, *10*(2). 10.3390/healthcare10020291 PMC8871802 35206905

[CR35] Silveira, B., Silva, H. S., Murai, F., & da Silva, A. P. C. (2021). Predicting user emotional tone in mental disorder online communities. *Future Generation Computer Systems*, *125*, 641–651. 10.1016/j.future.2021.07.014

[CR36] Stamatis, C. A., Meyerhoff, J., Liu, T., Sherman, G., Wang, H., Liu, T., Curtis, B., Ungar, L. H., & Mohr, D. C. (2022). Prospective associations of text-message-based sentiment with symptoms of depression, generalized anxiety, and social anxiety. *Depression and Anxiety*, *39*, 794–804. 10.1002/da.23286 36281621 PMC9729432

[CR37] Suslow, T., Husslack, A., Kersting, A., & Bodenschatz, C. M. (2020). Attentional biases to emotional information in clinical depression: A systematic and meta-analytic review of eye tracking findings. *Journal of Affective Disorders*, *274*, 632–642. 10.1016/j.jad.2020.05.140 32663997

[CR39] Tuck, A. B., Long, K. A., & Thompson, R. J. (2023). Social media’s influence on momentary emotion based on people’s initial mood: An experimental design. *Cognition and Emotion*, *37*(5), 1049–1056. 10.1080/02699931.2023.2219443 37265112

[CR40] Tuck, A. B., Jackson, J. J., & Thompson, R. J. (2025). College student depressive symptoms linked to feeling worse during social media use and engaging in social media in more emotionally negative ways: An experimental approach. *Emotion*. 10.1037/emo0001558. Advance online publication. 40549626

[CR41] Vandendriessche, H., Demmou, A., Bavard, S., Yadak, J., Lemogne, C., Mauras, T., & Palminteri, S. (2023). Contextual influence of reinforcement learning performance of depression: evidence for a negativity bias? *Psychological Medicine*, *53*(10), 4696–4706. 10.1017/S0033291722001593 35726513

[CR42] Villarroel, M. A., & Terlizzi, E. P. (2020). Symptoms of depression among adults: United States, 2019. NCHS Data Brief, no 379. National Center for Health Statistics. 33054920

[CR43] Watson, D., Clark, L. A., & Tellegen, A. (1988). Development and validation of brief measures of positive and negative affect: The PANAS scales. *Journal of Personality and Social Psychology*, *54*(6), 1063–1070. 10.1037/0022-3514.54.6.1063 3397865

[CR44] Watson, D., Weber, K., Assenheimer, J. S., Clark, L. A., Strauss, M. E., & McCormick, R. A. (1995). Testing a tripartite model: I. Evaluating the convergent and discriminant validity of anxiety and depression symptom scales. *Journal of Abnormal Psychology*, *104*(1), 3. 10.1037/0021-843X.104.1.3 7897050

[CR45] Wolters, N. E., Mobach, L., Wuthrich, V. M., Vonk, P., Van der Heijde, C. M., Wiers, R. W., Rapee, R. M., & Klein, A. M. (2023). Emotional and social loneliness and their unique links with social isolation, depression and anxiety. *Journal of Affective Disorders*, *329*, 207–217. 10.1016/j.jad.2023.02.096 36842647

[CR46] Zhang, X., Yu, H. W., & Barrett, L. F. (2014). How does this make you feel? A comparison of four affect induction procedures. *Frontiers in Psychology*, *5*, 689. 10.3389/fpsyg.2014.00689 25071659 PMC4086046

