# Supplementary material for: Depressive Symptoms Are Associated with Linguistic Features of Negative but Not Positive Autobiographical Narratives
Source: Affect Sci. 2026 Aug 11;7(3):464–74. doi: 10.1007/s42761-026-00395-4 (PMC13582712; doi:10.1007/s42761-026-00395-4)
Supplement: Supplementary file 1 [file 42761_2026_395_MOESM1_ESM.docx]

**Supplemental Materials**

**Associations Between Depressive Symptoms and Linguistic Features of Positive and Negative Autobiographical Narratives**

Alison B. Tuck, Yiqin Zhu, & Renee J. Thompson

Washington University in St. Louis, Department of Psychological & Brain Sciences

CB 1125

One Brookings Drive

St. Louis, MO 63130-4899

**Table S1.** Linguistic features predicted by severity of depression, induction conditions (positive vs. negative), and their interaction (*N* = 460; retaining responses over 25 words)

| Linguistic Feature | Predictors | Hypothesis | Results | | | | |
| --- | --- | --- | --- | --- | --- | --- | --- |
|  |  | Direction of effect | R^2^ | *b* | 𝛽 | 95% CI of 𝛽 | *p* |
| Positive Tone | **Intercept** | **n/a** | **.40** | **1.88** | **n/a** | **n/a** | **.003** |
|  | **Condition** | **Positive > Negative** |  | **4.59** | **0.84** | **[ 0.51, 1.17]** | **< .001** |
|  | Depression (Condition=negative) | ***ß*_Neg_** < 0 |  | 0.0004 | 0.002 | [-0.10, 0.10] | 0.964 |
|  | Depression (Condition=positive) | *ß*_Pos_ < 0 |  | -0.02 | -0.09 | [-0.19, 0.01] | 0.092 |
|  | Depression*Condition | ***ß*_Neg_** < *ß*_Pos_ < 0 |  | -0.02 | -0.22 | [-0.55, 0.12] | 0.207 |
| Negative Tone | **Intercept** | **n/a** | **.45** | **2.16** | **n/a** | **n/a** | **<.001** |
|  | **Condition** | **Positive < Negative** |  | **-1.54** | **-0.33** | **[-0.65, -0.02]** | **0.040** |
|  | **Depression (Condition=negative)** | ***ß*_Neg_ > 0** |  | **0.03** | **0.16** | **[ 0.06, 0.25]** | **0.001** |
|  | Depression (Condition=positive) | *ß*_Pos_ > 0 |  | 0.002 | 0.01 | [-0.09, 0.12] | 0.779 |
|  | **Depression*Condition** | ***ß*_Neg_ > *ß*_Pos_ > 0** |  | **-0.02** | **-0.34** | **[-0.66, -0.01]** | **0.040** |
| I-usage | **Intercept** | **n/a** | **.09** | **7.95** | **n/a** | **n/a** | **<.001** |
|  | Condition | Positive < Negative |  | 1.12 | 0.14 | [-0.27, 0.54] | 0.511 |
|  | **Depression (Condition=negative)** | ***ß*_Neg_ > 0** |  | **0.04** | **0.14** | **[ 0.02, 0.26]** | **0.025** |
|  | Depression (Condition=positive) | *ß*_Pos_ < 0 |  | -0.01 | -0.05 | [-0.18, 0.08] | 0.475 |
|  | **Depression*Condition** | ***ß*_Pos_ < 0 < *ß*_Neg_** |  | **-0.05** | **-0.44** | **[-0.85, -0.02]** | **0.041** |
| Affiliation Words | **Intercept** | **n/a** | **.03** | **3.49** | **n/a** | **n/a** | **<.001** |
|  | **Condition** | **Positive > Negative** |  | **2.35** | **0.43** | **[0.01, 0.85]** | **.047** |
|  | Depression (Condition=negative) | ***ß*_Neg_** < 0 |  | -0.002 | -0.01 | [-0.13, 0.12] | .899 |
|  | Depression (Condition=positive) | *ß*_Pos_ < 0 |  | -0.02 | -0.13 | [-0.26, 0.01] | .064 |
|  | Depression*Condition | ***ß*_Neg_** = *ß*_Pos_ |  | -0.02 | -0.28 | [-0.71, 0.15] | .204 |

**Table S2.** Linguistic features predicted by severity of depression, induction conditions (positive vs. negative), and their interaction (*N* = 453; retaining responses over 40 words) when controlling for age

| Linguistic Feature | Predictors | Hypothesis | Results | | | | |
| --- | --- | --- | --- | --- | --- | --- | --- |
|  |  | Direction of effect | R^2^ | *b* | 𝛽 | 95% CI of 𝛽 | *p* |
| Positive Tone | Intercept | n/a | .41 | -1.48 | n/a | n/a | .41 |
|  | **Condition** | **Positive > Negative** |  | **4.64** | **0.86** | **[0.53, 1.19]** | **< .001** |
|  | Depression (Condition=negative) | ***ß*_Neg_** < 0 |  | 0.001 | 0.006 | [-0.09, 0.10] | 0.899 |
|  | Depression (Condition=positive) | *ß*_Pos_ < 0 |  | -0.02 | -0.10 | [-0.20, 0.01] | 0.072 |
|  | Depression*Condition | *ß*_Neg_ < *ß*_Pos_ < 0 |  | -0.02 | -0.24 | [-0.58, 0.10] | 0.161 |
|  | Age | n/a |  | 0.17 | 0.07 | [0.00, 0.15] | 0.048 |
| Negative Tone | **Intercept** | **n/a** | **.45** | **3.10** | **n/a** | **n/a** | **0.038** |
|  | **Condition** | **Positive < Negative** |  | **-1.52** | **-0.33** | **[-0.65, -0.01]** | **0.043** |
|  | **Depression (Condition=negative)** | ***ß*_Neg_ > 0** |  | **0.03** | **0.16** | **[0.07, 0.26]** | **<0.001** |
|  | Depression (Condition=positive) | *ß*_Pos_ > 0 |  | 0.003 | 0.02 | [-0.08, 0.12] | 0.754 |
|  | **Depression*Condition** | ***ß*_Neg_ > *ß*_Pos_ > 0** |  | **-0.02** | **-0.34** | **[-0.67, -0.01]** | **0.041** |
|  | **Age** | **n/a** |  | **-0.05** | **-0.02** | **[-0.09, 0.04]** | **0.486** |
| I-usage | **Intercept** | **n/a** | **.09** | **8.04** | **n/a** | **n/a** | **0.043** |
|  | Condition | Positive < Negative |  | 1.20 | 0.15 | [-0.26, 0.56] | 0.482 |
|  | **Depression (Condition=negative)** | ***ß*_Neg_ > 0** |  | **0.04** | **0.14** | **[0.02, 0.26]** | **0.021** |
|  | Depression (Condition=positive) | *ß*_Pos_ < 0 |  | -0.01 | -0.05 | [-0.18, 0.08] | 0.469 |
|  | **Depression*Condition** | ***ß*_Pos_ < 0 < *ß*_Neg_** |  | **-0.06** | **-0.45** | **[-0.87, -0.03]** | **0.036** |
|  | Age | n/a |  | 0.06 | 0.02 | [-0.07, 0.10] | 0.735 |
| Affiliation Words | Intercept | n/a | .03 | 3.23 | n/a | n/a | 0.168 |
|  | **Condition** | **Positive > Negative** |  | **2.38** | **0.42** | **[0.002, 0.85]** | **0.049** |
|  | Depression (Condition=negative) | ***ß*_Neg_** < 0 |  | -0.002 | -0.01 | [-0.13, 0.12] | 0.892 |
|  | Depression (Condition=positive) | *ß*_Pos_ < 0 |  | -0.02 | -0.13 | [-0.26, 0.01] | 0.064 |
|  | Depression*Condition | ***ß*_Neg_** = *ß*_Pos_ |  | -0.02 | -0.28 | [-0.71, 0.15] | 0.208 |
|  | Age | n/a |  | 0.01 | 0.006 | [-0.09, 0.10] | 0.896 |

**Table S3.** Linguistic features predicted by severity of depression, induction conditions (positive vs. negative), their interaction, and changes in NA and PA (*N* = 453; retaining responses over 40 words)

| Linguistic Feature | Predictors | Model 1 Results |  | Model 2 Results |  |
| --- | --- | --- | --- | --- | --- |
|  |  | *b* [*SE*] | *p* | *b* [*SE*] | *p* |
| Negative Tone | Condition | **-1.55 [0.73]** | **.034** | -1.36 [0.74] | .066 |
|  | **Depression** | **0.03 [0.01]** | **.001** | **0.03 [0.01]** | **.001** |
|  | **Depression*Condition** | **-0.02 [0.01]** | **.042** | **-0.02 [0.01]** | **.043** |
|  | NA changes | / | / | 0.02 [0.02] | .178 |
|  | PA changes | / | / | -0.01 [0.01] | .421 |
|  |  |  |  |  |  |
| I-usage | Condition | 1.19 [1.71] | .486 | 1.05 [1.73] | .544 |
|  | **Depression** | **0.04 [0.02]** | **.023** | **0.04 [0.02]** | **.020** |
|  | **Depression*Condition** | **-0.05 [0.03]** | **.038** | **-.05 [0.03]** | **.039** |
|  | NA changes | **/** | **/** | 0.04 [0.04] | .335 |
|  | PA changes | **/** | **/** | 0.05 [0.03] | .123 |

*Note.* NA = Negative Affect; PA = Positive Affect; SE = Standard Error. Significant findings are bolded.
